# Investigating genomic prediction strategies for grain carotenoid traits in a tropical/subtropical maize panel

**DOI:** 10.1093/g3journal/jkae044

**Published:** 2024-03-01

**Authors:** Mary-Francis LaPorte, Willy Bayuardi Suwarno, Pattama Hannok, Akiyoshi Koide, Peter Bradbury, José Crossa, Natalia Palacios-Rojas, Christine Helen Diepenbrock

**Affiliations:** Department of Plant Sciences, University of California, Davis, Davis, CA 95616, USA; Department of Agronomy and Horticulture, Faculty of Agriculture, IPB University, Bogor 16680, Indonesia; Division of Agronomy, Faculty of Agricultural Production, Maejo University, Chiang Mai 50200, Thailand; Plant Breeding and Plant Genetics Program, University of Wisconsin-Madison, Madison, WI 53706, USA; Department of Plant Sciences, University of California, Davis, Davis, CA 95616, USA; United States Department of Agriculture-Agricultural Research Service, Robert W. Holley Center for Agriculture and Health, Ithaca, NY 14853, USA; International Maize and Wheat Improvement Center (CIMMYT), Km 45 Carretera Mexico-Veracruz, Texcoco 56130, Mexico; International Maize and Wheat Improvement Center (CIMMYT), Km 45 Carretera Mexico-Veracruz, Texcoco 56130, Mexico; Department of Plant Sciences, University of California, Davis, Davis, CA 95616, USA

**Keywords:** genomic prediction, maize, provitamin A, carotenoids, biofortification

## Abstract

Vitamin A deficiency remains prevalent on a global scale, including in regions where maize constitutes a high percentage of human diets. One solution for alleviating this deficiency has been to increase grain concentrations of provitamin A carotenoids in maize (*Zea mays* ssp. *mays* L.)—an example of biofortification. The International Maize and Wheat Improvement Center (CIMMYT) developed a Carotenoid Association Mapping panel of 380 inbred lines adapted to tropical and subtropical environments that have varying grain concentrations of provitamin A and other health-beneficial carotenoids. Several major genes have been identified for these traits, 2 of which have particularly been leveraged in marker-assisted selection. This project assesses the predictive ability of several genomic prediction strategies for maize grain carotenoid traits within and between 4 environments in Mexico. Ridge Regression-Best Linear Unbiased Prediction, Elastic Net, and Reproducing Kernel Hilbert Spaces had high predictive abilities for all tested traits (β-carotene, β-cryptoxanthin, provitamin A, lutein, and zeaxanthin) and outperformed Least Absolute Shrinkage and Selection Operator. Furthermore, predictive abilities were higher when using genome-wide markers rather than only the markers proximal to 2 or 13 genes. These findings suggest that genomic prediction models using genome-wide markers (and assuming equal variance of marker effects) are worthwhile for these traits even though key genes have already been identified, especially if breeding for additional grain carotenoid traits alongside β-carotene. Predictive ability was maintained for all traits except lutein in between-environment prediction. The TASSEL (Trait Analysis by aSSociation, Evolution, and Linkage) Genomic Selection plugin performed as well as other more computationally intensive methods for within-environment prediction. The findings observed herein indicate the utility of genomic prediction methods for these traits and could inform their resource-efficient implementation in biofortification breeding programs.

## Introduction

Vitamin A deficiency is a global issue, impacting approximately 30% of children less than 5 years old worldwide, that can cause xerophthalmia (conjunctival and corneal dryness), blindness (particularly night blindness), morbidity, and mortality, particularly for children and pregnant women (World Health Organization 2009, 2014; Wirth et al. 2017; Hodge and Taylor 2023). Deficiencies can be alleviated through increased intake of provitamin A carotenoids in the diet (Bouis and Welch 2010; Pixley et al. 2013; Prasanna et al. 2020). Biofortification is a multifaceted strategy to address malnutrition through the enhancement of nutritional quality in crops via breeding and/or agronomic practices (Bouis and Welch 2010). Biofortifying maize with higher grain concentrations of provitamin A carotenoids through breeding can help alleviate Vitamin A deficiency, especially in regions, including parts of sub-Saharan Africa, where a large portion of the daily diet typically comes from maize (*Zea mays*) (Bouis and Welch 2010; Pixley et al. 2013; Saltzman et al. 2013).

Ongoing biofortification efforts have resulted in lines with higher concentrations of provitamin A carotenoids (12–15 μg/g dry weight) than the average for maize grain (<1.5 μg/g dry weight) (Andersson et al. 2017; Ortiz et al. 2018). The International Maize and Wheat Improvement Center (CIMMYT, a member of CGIAR) developed and used a Carotenoid Association Mapping (CAM) panel of 380 maize inbred lines with different levels of grain carotenoids with growth phenotypes adapted to tropical and subtropical environments, which are relevant to the global target regions for carotenoid biofortification in sub-Saharan Africa and Latin America (Suwarno et al. 2015). The grain carotenoid traits of interest for the present study—β-carotene, β-cryptoxanthin, lutein, zeaxanthin, and provitamin A—were found to be highly heritable in the CAM panel in 3 environments, with broad-sense heritabilities ranging from 0.89 to 0.93 as reported by Suwarno et al. (2015). Ten lines included in the CAM panel have been biofortified (using marker-assisted selection; MAS) for an allele of *crtRB1 (β-carotene hydroxylase 1)* previously found to be associated with higher levels of provitamin A carotenoids (Yan et al. 2010; Babu et al. 2013; Suwarno et al. 2015).

Provitamin A and other health-beneficial carotenoids are synthesized in maize grain, primarily in the endosperm (Blessin et al. 1963; Weber 1987). Carotenoids are C40 isoprenoids, which can be classified as carotenes (including β-carotene) and xanthophylls (oxygenated carotenoids, including β-cryptoxanthin, zeaxanthin, and lutein). β-Carotene and β-cryptoxanthin are the provitamin A carotenoids included in this study. Provitamin A is a derived trait, calculated based on retinol equivalents (Babu et al. 2013, Suwarno et al. 2015); i.e. provitamin A = β-carotene + 0.5(β-cryptoxanthin), due to β-carotene providing 2 units of retinol (vitamin A) upon oxidative cleavage in human and animal systems, whereas β-cryptoxanthin provides only 1 unit. Certain *cis* isomers of β-carotene, namely 9-*cis*-β-carotene and 13-*cis*-β-carotene, have also been of specific interest (to be quantified individually) for human nutrition given that their concentration may change during cooking, and they may have different bioavailability (compared to all-*trans*-β-carotene) during the digestion process (Bohn et al. 2019). Lutein and zeaxanthin are not provitamin A carotenoids, but are nonetheless crucial to human health, specifically eyesight and eye development (Krinsky et al. 2003; Bernstein and Arunkumar 2021). Zeaxanthin is the most abundant grain carotenoid in the population under study herein (Suwarno et al. 2015).

Genomic prediction (GP) methods use genetic markers located throughout the genome to estimate breeding values for phenotypic traits (Crossa et al. 2017). GP can be a valuable tool to accelerate genetic gain in a breeding program by increasing the accuracy of selections and/or reducing cycle time (Crossa et al. 2017). In their review of the implementation of GP in CIMMYT maize and wheat breeding programs, Crossa et al. (2014) described 2 primary applications: predicting breeding values of individuals for rapid cycling, and predicting genotypic (including epistatic) values of advanced breeding lines; the present study will focus on the former, given the structure of the CAM panel involved herein.

Genomic prediction is typically deployed in 1 or multiple of 4 scenarios: (1) tested lines are predicted in tested environments; (2) tested lines are predicted in untested environments; (3) untested lines are predicted in tested environments; and (4) untested lines are predicted in untested environments. GP allows the researcher to leverage the known associations between genetic markers and the phenotype in lines from (2)–(4) to make informed predictions. Within-environment prediction is a scenario in which the model is trained on lines grown in a particular environment (location and year) and used to predict the performance of lines grown in that same environment. In between-environment prediction, a model that has been trained on lines grown in 1 environment is used to predict breeding values for grain carotenoid traits of lines that were grown in a different geographic location and/or year than the lines included in the training set. Predicting the breeding values for 1 or more phenotypes of interest of lines in an unknown environment using GP could be expected to work best for traits that are highly heritable in the respective target population of environments because the environment has less of an impact on the phenotype.

GP strategies targeting genome-wide markers or markers proximal to 8 or 58 a priori genes were previously found to exhibit high prediction accuracy for a similar set of grain carotenoid traits (Owens et al. 2014) in 201 inbred lines with yellow to orange grain from a maize diversity panel (Flint-Garcia et al. 2005). That panel consisted primarily of lines with temperate adaptation, with only approximately one-quarter of the lines having tropical/subtropical adaptation (both in the original set of 302 lines and in the 201-line subset analyzed in Owens et al. 2014), and was grown in Indiana, in the Midwest region of the United States (Owens et al. 2014). Testing of multiple marker sets and regression methods in a tropical/subtropical panel that was grown in the corresponding target environments would be informative in assessing the utility of GP for tropical/subtropical biofortification breeding programs in which MAS has been deployed. The high heritabilities of the carotenoid traits in the CAM panel in tested tropical/subtropical environments (as reported in Suwarno et al. 2015) make them good candidates for GP given that heritability represents the proportion of phenotypic variance that is explained by genetic factors. Implementation of GP strategies has been recently explored to support the biofortification of vitamin E (Tibbs-Cortes et al. 2022) and zinc-related (Guo et al. 2020) traits in maize, iron and zinc (among other health-beneficial mineral nutrients) in wheat (Velu et al. 2016, Manickavelu et al. 2017, Joukhadar et al. 2021), and zinc in rice (Rakotondramanana et al. 2022).

Several genes have been identified through genome-wide association studies (GWAS) for grain concentrations of provitamin A and other carotenoids in maize (Harjes et al. 2008; Yan et al. 2010; Owens et al. 2014; Suwarno et al. 2015; Azmach et al. 2018; Baseggio et al. 2020, Diepenbrock et al. 2021; LaPorte et al. 2022), and could inform the marker sets to be targeted for use in GP. Two of these genes have been selected upon via MAS: *crtRB1 (β-carotene hydroxylase 1)*, which catalyzes the hydroxylation of β-carotene to β-cryptoxanthin to zeaxanthin, and *lcyE (lycopene ε-cyclase)*, which catalyzes ε-ring cyclization at the branchpoint of the core carotenoid pathway (Yan et al. 2010; Babu et al. 2013; Suwarno et al. 2015). A larger set of 13 genes was identified for grain carotenoid traits in the US maize nested association mapping panel (Diepenbrock et al. 2021; LaPorte et al. 2022; Supplementary Table 1), for which approximately half of the parents were tropical in adaptation.

Three types of regression have been extensively used to train GP models (Usai et al. 2009; Heslot et al. 2012; Azodi et al. 2019): Ridge Regression-Best Linear Unbiased Prediction (RR-BLUP; Meuwissen et al. 2001; Endelman 2011), Least Absolute Shrinkage and Selection Operator (LASSO; Tibshirani 1996), and Elastic Net (Zou and Hastie 2005). These methods are regularization techniques, meaning that they penalize model complexity and differ based on their penalization strategies (Ogutu et al. 2012). In brief, the penalization strategy for Ridge Regression (L2 regularization) is the sum of squares of the coefficients of the model. By contrast, LASSO (L1 regularization) uses the absolute value of the coefficients and will zero coefficients for a sparse solution. Elastic Net is an intermediate method that utilizes both L1 and L2 regularization, modulated by a hyperparameter, *α* (Zou and Hastie 2005). Models that conduct feature selection, such as LASSO or EN, may be advantageous for traits with relatively oligogenic architectures (Heslot et al. 2012, de los Campos et al. 2013). A Reproducing Kernel Hilbert Space (RKHS)-based approach has been developed for GP to take advantage of semi-supervised learning based on a projection into a Hilbert space (which is a mathematical construct that allows for such a projection) to model nonlinear relationships between predictors and traits more accurately (Gianola et al. 2006; de los Campos et al. 2010; Montesinos López et al. 2022). Kernel approaches, including RKHS, theoretically will have higher predictive abilities for traits with a more complex genetic architecture, that cannot be described by a linear model, underpinning the phenotype (Montesinos López et al. 2022).

Implementation of GP relies on similar datasets as those used for genetic mapping—i.e. genotypic and phenotypic data. As such, being able to conduct GP in the same analytical workflow as genetic mapping and other quantitative genetics analyses could offer benefits from a computational efficiency perspective and alleviate barriers to entry for GP approaches that otherwise require custom scripting (and, for some implementations, high-performance computing). A software package called Trait Analysis by aSSociation, Evolution and Linkage (TASSEL; Bradbury et al. 2007) is widely used among the plant breeding/genetics community for quantitative genetics analyses. TASSEL is well documented and maintained, can be operated in a graphical user interface (which is also helpful for alleviating barriers to entry), and is configured to accept multiple common formats of genotypic and phenotypic data.

This study investigates the predictive ability of several GP models to inform the implementation of genomic prediction/selection (GP/GS) in breeding efforts for carotenoid-dense tropical and subtropical maize. We compared several GP methods, targeting genome-wide markers with and without feature selection vs markers proximal to 2 or 13 genes, to determine the method with the highest predictive ability for grain carotenoid traits within environments. Regression types tested within environments included RR-BLUP, GBLUP implemented in the TASSEL Genomic Selection plugin, LASSO, Elastic Net, and RKHS. Additionally, we tested the efficacy of RR-BLUP to predict between environments. Furthermore, we compared the predictive abilities of models trained on genome-wide markers vs markers proximal to 2 genes (*crtRB1* and *lcyE*) or 13 genes (Supplementary Table 1) of known relevance to grain carotenoid biosynthesis within and between environments. Finally, we describe the performance of a Genomic Selection plugin that has been implemented in TASSEL for use by researchers and test the predictive ability and computational efficiency of this plugin, including in comparison with other methods.

## Methods

### Maize germplasm

The design of the CAM panel was described in Suwarno et al. (2015). Briefly, inbred lines were selected at the initial stages of the CIMMYT-HarvestPlus maize biofortification breeding program, more than a decade ago, for inclusion in the panel. The CAM panel includes 380 lines, with the majority being tropical or subtropical lines (47% each) and 3% of lines adapted to temperate conditions (Suwarno et al. 2015). In addition to the CAM panel, 65 other CIMMYT lines were quantified in the same environments and were included in GP. These lines were distributed throughout the population in terms of genomic relatedness (Supplementary Fig. 1). Of the included maize lines, 10 lines in this study include the beneficial alleles of one gene associated with high provitamin A (*crtRB1*). The pedigree of these lines was reported by Suwarno et al. (2015). These lines were: CIM-SYN-386-34, CIM-SYN-387-35, CIM-SYN-389-37, CIM-SYN-390-38, CIM-SYN-391-39, CIM-SYN-392-40, CIM-SYN-393-41, CIM-SYN-394-42, CIM-SYN-415-63, and CIM-SYN-416-64. The CAM panel was grown in Agua Fría (AF) in the state of Puebla, Mexico, in the years 2012 and 2013, and Tlaltizapan (TL) in the state of Morelos, Mexico, in the years 2010 and 2011, resulting in 4 environments hereafter referred to as the combination of location and year: TL10, TL11, AF12, and AF13 (Suwarno et al. 2015; Supplementary Fig. 2). Tlaltizapan (which is considered mid-altitude subtropical) is located 268 km south and west of Agua Fría (which is considered tropical). Tlaltizapan had, at the time of experimentation, a slightly warmer (+1.5°C), drier (−360 mm/year) climate at a higher elevation (+835 masl) than Agua Fría (Suwarno et al. 2015). Tlaltizapan and Agua Fría are in Aw (class 3) and Am (class 2), respectively, according to the Köppen–Geiger classification (Beck et al. 2018; Supplementary Fig. 2).

### Phenotypic datasets

Lines from the 4 distinct environments (AF12, AF13, TL10, TL11) were phenotyped for the following traits: lutein, zeaxanthin, β-carotene, β-cryptoxanthin, and provitamin A [defined as the concentration of β-carotene + 0.5(β-cryptoxanthin)] as described in Suwarno et al. (2015). In the Agua Fría environments, lines were also phenotyped for 2 isomers of β-carotene: 9-*cis*-β-carotene and 13-*cis*-β-carotene. Briefly, 2–6 plants per plot were self-pollinated, and random samples of 50 seeds from each accession were ground and quantified for carotenoid analysis following the CIMMYT laboratory procedure (Galicia et al. 2009; Palacios-Rojas et al. 2017). In samples collected from TL10 and TL11, concentrations of individual carotenoid compounds were quantified using HPLC and in Agua Fría, they were quantified using UPLC (Suwarno et al. 2015). The results were reported as μg g^−1^ dry kernel weight (Galicia et al. 2009; Suwarno et al. 2015).

### Data preparation

Genotypic data for each accession were generated as described by Suwarno et al. (2015). Briefly, genotyping by sequencing (GBS) (955,120 SNPs) was used for these lines, as generated by the Institute for Genomic Diversity, Cornell University, Ithaca, NY, USA (Suwarno et al. 2015). Filtering for minor allele frequencies (MAF) was not conducted before GP. The genotypic data, in the form of a matrix of SNP markers (including monomorphic markers), were numericalized using the HapMap() function from Genome Association and Prediction Integrated Tool (GAPIT) version 3 in R (R Core Team 2021; Wang and Zhang 2021) for all methods except for the TASSEL plugin, for which a kinship matrix was calculated within TASSEL from the input genotypic dataset as described below. The phenotypic data for each line and trait were matched to the genotype using the accession identifier. Data are available at: https://hdl.handle.net/11529/10549016 (LaPorte et al. 2024).

### Model types

The model types used in the model fitting procedure described above include RR-BLUP, Least Absolute Shrinkage and Selection Operator (LASSO), Elastic Net (EN), and Reproducing Kernel Hilbert Spaces (RKHS). RR-BLUP was completed using the rrBLUP package (Endelman 2011) function kinship.BLUP(). This function carries out both the training and prediction steps. The median and standard deviation (SD) of predictive ability across the 5 folds were calculated and reported. LASSO was fit using the GLMNet package (Friedman et al. 2010) function cv.glmnet(), with *α* = 1 and by tuning the lambda hyperparameter (the shrinkage parameter) by identifying the value with the lowest mean cross-validated error (referenced in the documentation as “cvm”) from a set of 250 potential values for each fold and iteration, using the nlambda argument (Friedman et al. 2010). Predictions were calculated using the GLMNet library function predict(). Within each cross-validation model fitting instance, Elastic Net was conducted 9 times using the GLMNet() function from the GLMNet package (Friedman et al. 2010), with a different *α* value each time. The 9 *α* values (the weight of L1 vs L2 regularization penalties) tested ranged from 0.1 through 0.9 with steps of 0.1. Note that an *α* value of 1 is equivalent to the LASSO method, and an *α* value equal to 0 corresponds to RR-BLUP in this function. The lambda (shrinkage) parameter was selected from a set of 250 values for each *α* value of Elastic Net separately, in the same manner as described above for LASSO.

The BGLR (Bayesian Generalized Linear Regression) function from the BGLR package was used to train and fit a Reproducing Kernel Hilbert Space (RKHS) model (Perez and de los Campos 2014). The Gaussian kernel was used as the covariance matrix (Kernel “K”), calculated using a bandwidth parameter of 1 and a distance matrix, D, containing the Euclidean distance between the training and test set of marker genotypes (a square matrix with the dimensions of the number of lines). We ran 6,000 iterations per fit model with a burn-in size of 1,000 (based on hyperparameter tuning with smaller burn-in sizes). The bglr() function was used to fit the model and generate the predicted values.

GBLUP, which is mathematically equivalent to RR-BLUP (Meuwissen et al. 2001; Habier et al. 2007; Clark and Van Der Werf 2013), was conducted using the TASSEL GUI built-in implementation (Bradbury et al. 2007). After genotypic data was loaded, a kinship matrix was created using TASSEL. Genomic prediction was conducted by selecting the phenotypic file and kinship matrix. Five-fold cross-validation was repeated in 20 iterations for each trait and environment. While all other methods (Ridge, EN, LASSO, RKHS) used the same line assignments for the training and test sets, TASSEL did not, as the plugin conducts random fold assignments unless given an incomplete phenotype vector (in which values in the desired test set have been masked). The RKHS, LASSO, and RR-BLUP methods were run with the same numericalization strategy and training and test splits (within a given iteration) and are thereby a direct comparison within a given trait. Running TASSEL on the same train–test-split assignments as the other methods was performed for a subset of iterations to determine the extent to which RR-BLUP predicted values were similar to those generated by TASSEL. RR-BLUP, RKHS, LASSO, and EN were run on a High-Performance Computing (HPC) cluster, with 250 Gb of memory allocated to a parallelized script. The TASSEL GUI and the 2-gene and 13-gene R scripts were all run on the desktop. The TASSEL software was run with a maximum heap of -Xmx 100g.

We also compared the use of subsets of known carotenoid-related genes in prediction to whole-genome prediction. Two-gene and 13-gene approaches were conducted with RR-BLUP, both within and between environments, using the markers within 250 kilobases (kb) of genes that are related to grain carotenoid traits (Supplementary Table 1); these markers were extracted using a custom R script. The locations of the genes used in the 2-gene and 13-gene approaches were determined using MaizeGDB (Woodhouse et al. 2021; Supplementary Table 1). The 2-gene approach included all markers within ±250 kb of *lcyE* and *crtRB1* (1,540 markers). The 13-gene approach included markers within ±250 kb of 13 identified carotenoid-related genes (11,143 markers) (Diepenbrock et al. 2021; LaPorte et al. 2022; Supplementary Table 1), which included *lcyE* and *crtRB1*.

Between-environment analyses were conducted using RR-BLUP. Every pairwise combination of environments (referenced here as environments E1 and E2) was evaluated as training and test sets for prediction. Five-fold cross-validation was conducted for each pair of environments E1 and E2, such that both within- and between-environment predictions were conducted in an identical framework. Namely, for a given fold of cross-validation, (1) 80% of E1 was used as the training set, and the remaining 20% of E1 was used as the test set; (2) 80% of E2 was used as the training set, and the remaining 20% of E2 was used as the test set; (3) 80% of E1 was used as the training set, and the remaining 20% of E2 was used as the test set; and finally, (4) 80% of E2 was used as the training set, and the remaining 20% of E1 was used as the test set.

Genomic prediction (GP) was conducted using a 5-fold cross-validation approach with 20 iterations. As a result, each model was run 100 times. For each cross-validation instance, the accessions were divided into randomized training and testing sets, with ∼80% of the lines used for training and the remaining ∼20% withheld for testing model performance. Each GP model was trained using genomic and phenotypic data in the training set, and the resulting model was then fit to the test set to predict their breeding values based on genomic information. Predictive ability for every model type was calculated as the Pearson correlation (*r*) between the predicted values for the test set and the actual observed values in the test set. The median predictive ability of each iteration was calculated and reported. The 20-iteration, 5-fold cross-validation process was repeated with each model-fitting method (RR-BLUP, EN, LASSO, and RKHS) for each of the carotenoid traits within each environment, using the same lines in the training and test sets for each method. For within-environment GP, the training and test sets were grown in the same environment (AF12, AF13, TL10, TL11). The 5-fold cross-validation process was also conducted with RR-BLUP between each environment for each of the carotenoid traits (β-carotene, β-cryptoxanthin, lutein, zeaxanthin, and provitamin A in all environments, as well as 9-*cis*-β-carotene and 13-*cis*-β-carotene in the Agua Fría environments).

Medians were depicted in the figures as a measure of central tendency for predictive ability because they are less affected by outliers than means. Plots for all models were visualized using ggplot2 and color palettes from Viridis and base R (Wickham 2016; R Core Team 2021; Garnier et al. 2023), and boxplots were ordered by median using the reorder() function from the R stats package (included in base R). To aid in figure readability, the results of the best-performing EN model [determined using the reorder() function across all traits and environments] were depicted in the main text, and the remainder of the EN results were reported in the supplementary. Models are systematically referred to as “similar to” and “different from” one another based on the differences in median predictive ability between all iterations for a given model type, trait, and environment. Namely, “similar to” refers to models where the median predictive ability across all iterations of one method was within the interquartile range (IQR) of predictive abilities of another method. Models being “better than” (or “worse than”) one another is defined as when the median for the “better” (or worse) method is higher (or lower) than the median of the second method and not within the IQR of that method.

## Results

### Phenotypic correlations

**Fig. 1.**
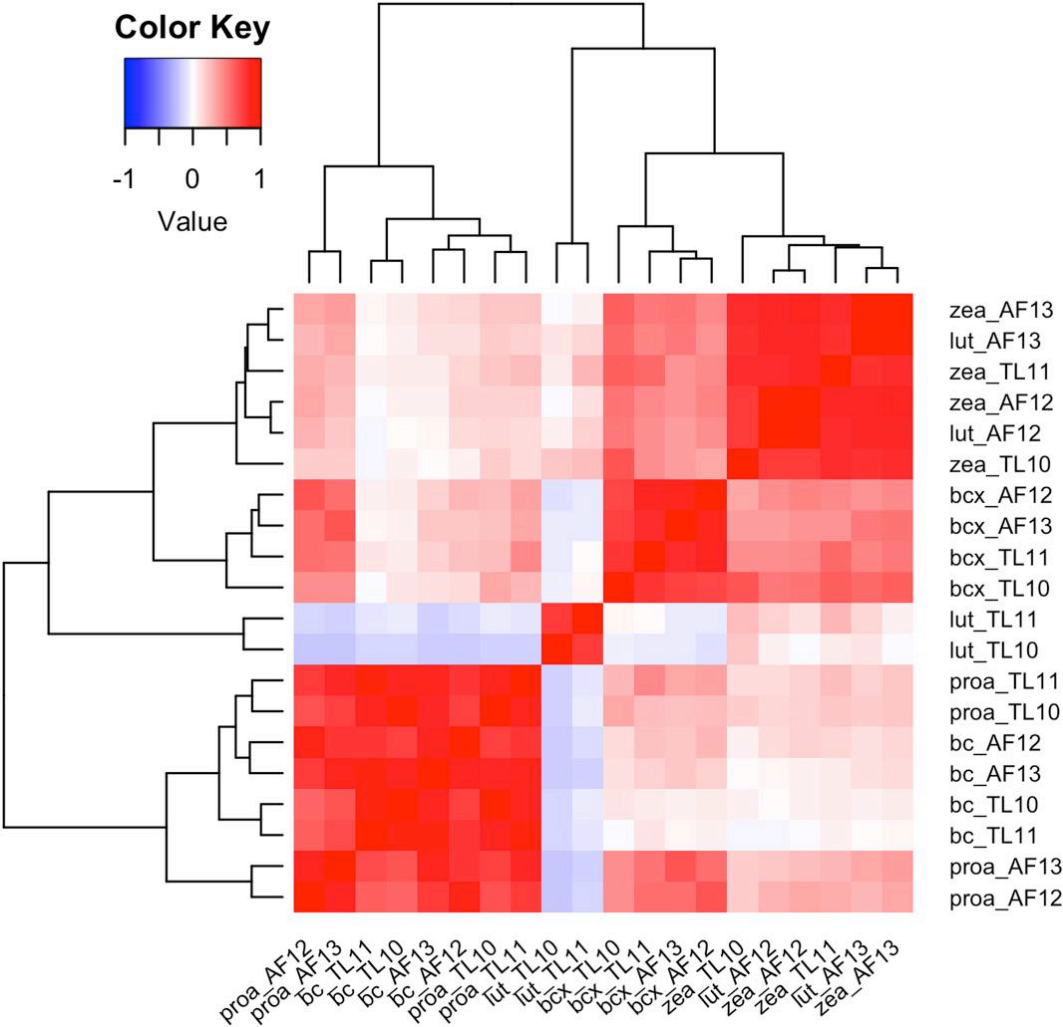
Correlations between phenotypic values for each trait and environment. The trait–environment pairs are listed as the abbreviation for the trait (lut, lutein; zea, zeaxanthin; bcx, β-cryptoxanthin; bc, β-carotene; proa, provitamin A) and the environment [Agua Fría 2012, Agua Fría 2013, Tlaltizapan 2010, and Tlaltizapan 2011 (AF12, AF13, TL10, and TL11, respectively)]. The heatmap is arranged in clustered groups denoted with the line diagram. Red corresponds to a positive correlation, and blue corresponds to a negative correlation. A darker color indicates a higher absolute value of the correlation.

The phenotypic values were positively correlated for most traits and in most environments (Fig. 1). Zeaxanthin levels in all environments and lutein in the Agua Fría environments were highly positively correlated. A high positive correlation was observed between provitamin A and β-carotene in all environments. A negative correlation was observed between lutein and each of provitamin A and β-carotene in the Tlaltizapan environments. β-Cryptoxanthin showed a slightly higher correlation with provitamin A within Agua Fría environments than within the Tlaltizapan environments.

### Within-environment prediction

**Fig. 2.**
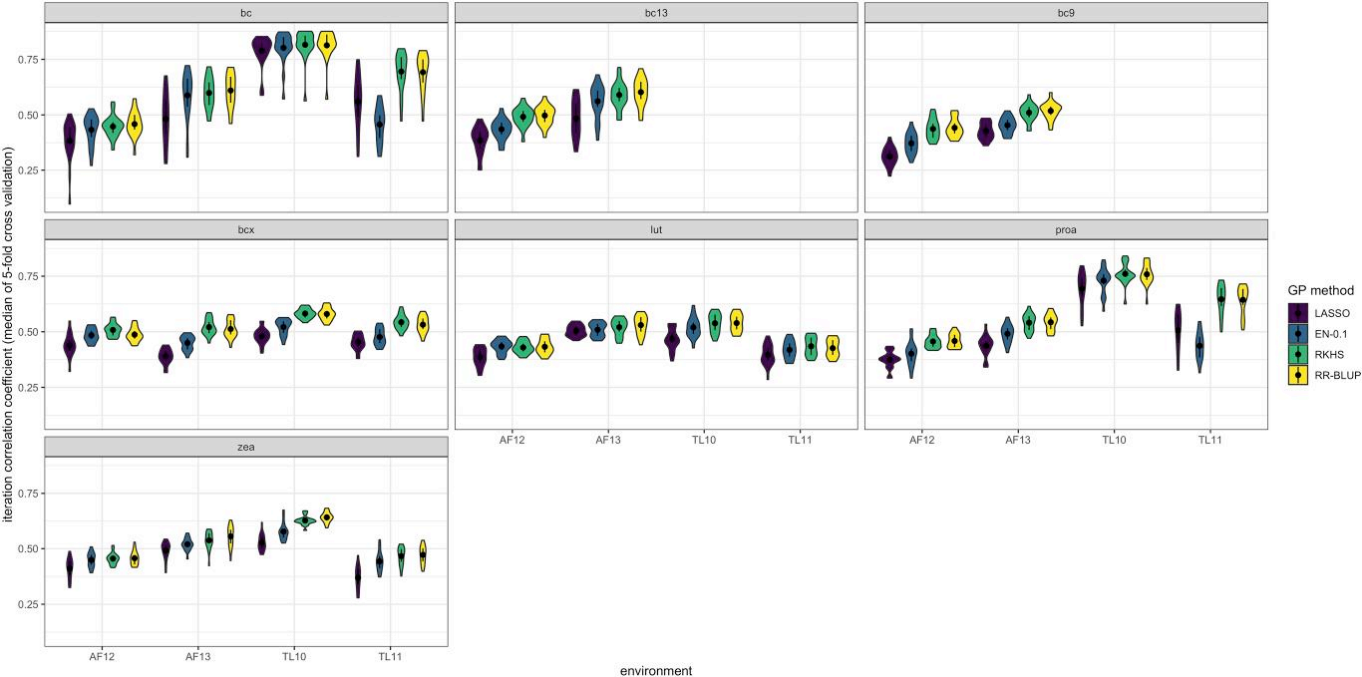
Within-environment predictive abilities for 4 regression types (RR-BLUP, EN, LASSO, and RKHS) for each trait–environment combination. RR-BLUP, Ridge Regression-Best Linear Unbiased Prediction; LASSO, Least Absolute Shrinkage and Selection Operator; EN, Elastic Net; RKHS, Reproducing Kernel Hilbert Space. For EN, the numeric value of 0.1 indicates the ɑ value. Trait abbreviations: bc, β-carotene; bc9, 9-*cis*-β-carotene; bc13, 13-*cis*-β-carotene; bcx, β-cryptoxanthin; lut, lutein; proa, provitamin A; zea, zeaxanthin. The median for each violin is represented by a black dot. The interquartile range (Q1–Q3) of each set of iterations is represented by a black vertical line through the center of each violin.

First, we compared the predictive abilities of RR-BLUP (*α* = 0), Elastic Net (*α* = 0.1–0.9), and LASSO (*α* = 1) (Fig. 2, Supplementary Fig. 3). For most traits and in most environments, RR-BLUP had the highest predictive ability, although in some cases, the predictive ability was similar to that of 1 or several EN models. Specifically, RR-BLUP had better predictive ability than the EN and LASSO methods for β-carotene (in TL11), 13-cis-β-carotene (in AF12), 9-cis-β-carotene (in AF12 and AF13), β-cryptoxanthin (in TL10, TL11, and AF13), provitamin A (in TL11, AF12, and AF13), and zeaxanthin (in TL10 and AF13). The EN model with *α* = 0.1 was generally the highest-performing EN model (Supplementary Fig. 3) and is therefore reported in Fig. 2 for comparison to RR-BLUP and LASSO. For most trait–environment combinations, LASSO performed similarly to the EN models except in the case of 3 traits in TL11. Specifically, LASSO performed worse than all EN models and RR-BLUP for zeaxanthin in TL11, and LASSO and RR-BLUP performed better than all of the EN models for β-carotene and provitamin A in TL11. High predictive abilities were observed for β-carotene and provitamin A in TL10 for all of RR-BLUP, EN, and LASSO, with the medians of all models higher than 0.75 for β-carotene and ranging from 0.71 to 0.75 for provitamin A. However, β-carotene and (to a lesser extent) provitamin A also generally had larger ranges of median predictive ability (represented by the height of the violins; Fig. 2) than the other traits in each environment. For this within-environment prediction experiment, RR-BLUP took the least computational time compared to EN and LASSO (which took approximately 4 times as long).

RR-BLUP and RKHS had similar predictive abilities for all traits and environments (Fig. 2). In all trait–environment combinations, both of these methods performed better than LASSO (which exhibited median predictive abilities of 0.10–0.59). Although there were some variations in the median predictive ability, none of the median predictive abilities for these 2 superior-performing methods were outside of the IQR of one another for a given trait–environment combination (0.32–0.88 for RR-BLUP, and 0.34–0.88 for RKHS). For RR-BLUP, the predictive ability was highest for β-carotene (highest median value of 0.831, for any trait–environment combination). Although the RKHS method performed as well as RR-BLUP in all instances, it took longer to run (approximately 6 times as long). RR-BLUP performed highly similarly to GBLUP conducted through the TASSEL Genomic Selection GUI plugin (Supplementary Fig. 4); these 2 methods are mathematically equivalent but used different fold assignments.

We plotted the median predictive ability for each iteration of the RR-BLUP within-environment prediction experiment vs the SD of predictive ability across folds within that iteration (Supplementary Fig. 5). No clear trend was observed between these 2 metrics for most traits and most environments. For certain traits and environments—for example, β-carotene in all environments—iterations with higher SD tended to have lower predictive ability. The raw phenotypic values were also examined to check for any relationship with SD of predictive ability within iterations; the range of phenotypic values was similar between the environments (Supplementary Fig. 6). The 10 biofortified lines in the panel under study herein had an allele of *crtRB1* that was previously found to be favorable for provitamin A (Yan et al. 2010; Babu et al. 2013; Suwarno et al. 2015). The lines that possessed these alleles indeed had among the highest levels of β-carotene and provitamin A (Supplementary Fig. 6). For the other traits, notably including β-cryptoxanthin, the phenotypic values were located throughout the population distribution.

### Between-environment prediction

**Fig. 3.**
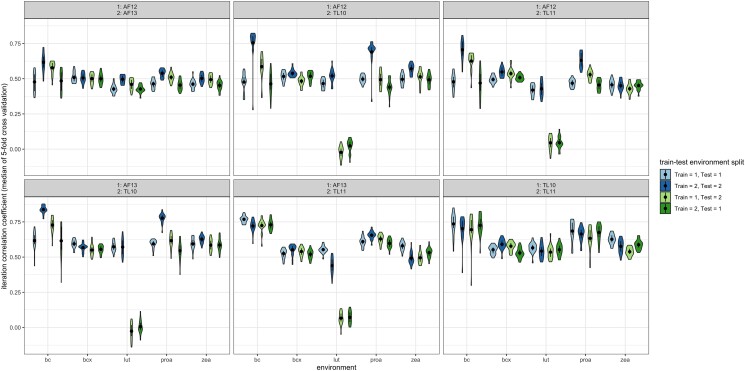
Between-environment predictive abilities using RR-BLUP for all traits, within every pairwise combination of environments. Trait abbreviations: bc, β-carotene; bc9, 9-*cis*-β-carotene; bc13, 13-*cis*-β-carotene; bcx, β-cryptoxanthin; lut, lutein; proa, provitamin A; zea, zeaxanthin.

RR-BLUP was performed between environments to test the extent to which the data from 1 environment could be used as a training set to predict values for a different environment. Between-environment predictions performed approximately as well as within-environment predictions for the lowest-predictive ability environment (Fig. 3). Notable exceptions include predictions for lutein in instances in which data from Agua Fría (AF) are being used to predict in Tlaltizapan (TL) and vice versa; in each of those cases, the predictive ability for lutein was near zero. The within-environment predictive abilities for TL10 for β-carotene and provitamin A were higher, and narrower in range (indicated by the height of the violin; Fig. 3), compared to the between-environment predictive abilities for TL10 when AF13 was used as the training set. When different years were compared in the same location (between AF12 and AF13, or between TL10 and TL11), the predictive abilities were similar to those of the across-location models. In some cases, there was a marginally higher predictive ability when an environment was used as the test rather than the training set. For example, higher predictive ability was observed for provitamin A when AF12 was used as the training set (0.531) and TL11 as the test set rather than the opposite scenario (0.456).

### Testing of marker sets

**Fig. 4.**
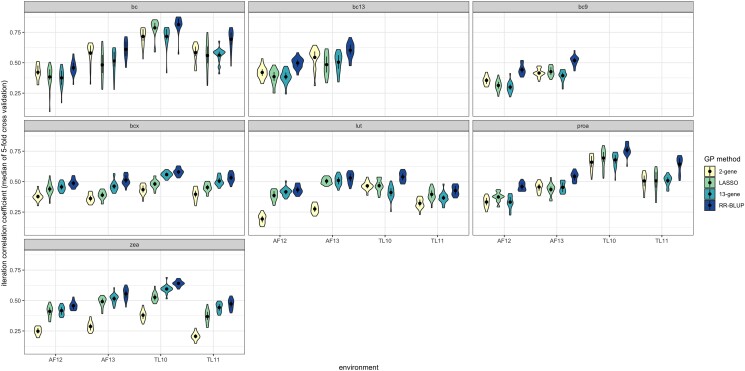
Comparison of predictive abilities for RR-BLUP and LASSO models using genome-wide markers to those of RR-BLUP models using markers proximal to 2 or 13 genes. Trait abbreviations: bc, β-carotene; bc9, 9-*cis*-β-carotene; bc13, 13-*cis*-β-carotene; bcx, β-cryptoxanthin; lut, lutein; proa, provitamin A; zea, zeaxanthin.

Next, we investigated if including fewer markers in RR-BLUP could have comparable predictive ability to RR-BLUP using genome-wide markers, which was the best-performing whole-genome prediction method (Fig. 4). For this purpose, we incorporated 2 additional approaches, referred to as the 2-gene and 13-gene approaches (Supplementary Table 1), that used a smaller subset of markers as the only predictors in the model (Fig. 4). The 2-gene and 13-gene RR-BLUP approaches both had lower predictive abilities than RR-BLUP using genome-wide markers. The 13-gene approach performed much better than the 2-gene approach for some trait–environment combinations: notably, lutein in the AF environments and zeaxanthin in all environments. The 13-gene approach performed better than LASSO (except for similar performance in AF12), which in turn performed better than the 2-gene approach, for β-cryptoxanthin. For β-carotene and provitamin A, the 2-gene and 13-gene approaches had similar predictive abilities. For 13-*cis*-β-carotene and 9-*cis*-β-carotene, the 2-gene method performed better than the 13-gene method. The 2- and 13-gene approaches maintained similar predictive abilities between vs within environments for all traits except for lutein, for which predictive abilities were near zero between locations (particularly for the 2-gene approach; Supplementary Figs. 7 and 8).

## Discussion

Genomic prediction is the central mathematical exercise in Genomic Selection. A key application of these models is the prediction of germplasm performance in tested and untested environments (Heslot et al. 2012, 2015; Bhat et al. 2016; Azodi et al. 2019). In this study, we predicted breeding values for maize grain carotenoid traits for tested lines in tested environments in 5-fold cross-validation and for those same lines in “untested” environments (for which phenotypes had been masked in 5-fold cross-validation) in between-environment prediction. Comparison of methods that use genome-wide markers in prediction (such as RR-BLUP) with those that conduct feature selection (such as LASSO and EN) or that only include markers proximal to 2 or 13 genes could inform optimal prediction strategies for these relatively oligogenic traits. Knowing the predictive ability of a model within and/or between environments is helpful for breeders who wish to use this model to predict the performance of tested and/or untested lines.

### Within-environment prediction

RR-BLUP consistently exhibited a higher predictive ability than LASSO. These results suggest that, in this dataset, penalizing model complexity and utilizing a smaller set of predictors was detrimental to model predictive ability, even for these grain carotenoid traits, which are thought to be relatively oligogenic in architecture. Incorporating more markers appears to be beneficial for model predictive ability within the model configurations tested within this study. This finding is corroborated by the results in Fig. 4, where the 2- and 13-gene approaches had lower predictive abilities than the RR-BLUP approach that included genome-wide markers. LASSO, which also tested a smaller subset of markers (that the model identifies through hyperparameter optimization) but which uses no prior information about the markers in the selection of the subset, performed worse than the 13-gene method (but better than the 2-gene method) for β-cryptoxanthin in 3 of 4 environments, and otherwise performed similarly to the 13-gene method (Fig. 4). In this study, whole-genome prediction methods had higher predictive abilities than methods that used marker subsets. The 2-gene approach was tested given the use of *lcyE* and *crtRB1* in MAS efforts specifically for provitamin A, whereas the 13-gene approach was tested to determine the predictive ability of a model focused on a broader set of a priori genes for grain carotenoid traits. Based on the 2-gene approach exhibiting worse predictive abilities than the 13-gene approach (and approaches using genome-wide markers) for zeaxanthin within each of the 4 environments, we particularly recommend using a marker set that is less targeted than the 2-gene subset for prediction of or selection upon zeaxanthin.

Previous literature suggests that it can be beneficial to include markers tagging 1–3 major genes or genomic regions (e.g. quantitative trait loci) of interest as fixed effects in genomic prediction (Rutkoski et al. 2012; Bernardo 2014; Spindel et al. 2016; Sehgal et al. 2020). To do so, it is necessary to identify the feature to be used in genomic prediction, such as a developed marker or haplotype representing the favorable allele. The former could be emulated by identifying a peak marker through a GWAS conducted on each training set in 5-fold cross-validation before the prediction step is conducted on the held-out fold (as in Spindel et al. 2016 and Rice and Lipka 2019), which was outside the scope of the present study. Further automation of that capability would be helpful for studies testing a large number of prediction scenarios. Bernardo (2014) found in simulation experiments that including fixed effects for major known genes is most beneficial when a trait is highly heritable and 1–3 major genes (and a marker tagging them) are identified. That finding suggests that GP approaches informed by GWAS in the training set may be beneficial if markers or haplotypes representing favorable alleles for *lcyE* and *crtRB1* are set as fixed effects, but the advantages of this approach vs using the deployed MAS markers (which have not been scored in the CAM panel) would need to be considered.

### Between-environment prediction

RR-BLUP was used for between-environment prediction because it had the highest predictive ability and computational efficiency of all regression methods (LASSO, EN, RKHS, Ridge) tested in the within-environment experiment (Figs. 1, 2, and 3). The between-environment models maintained predictive ability between locations and years (with the exception of lutein between locations), which is concordant with the high heritability of the tested traits, and suggests that the results of these predictions could be beneficial for breeders wishing to develop carotenoid-dense varieties for untested environments (at least within a similar climatic zone). Breeding of provitamin A-dense maize varieties is also taking place by teams at CIMMYT, IITA, and other institutions in southern and western Africa, among other geographies. Testing predictive ability in multi-environment trials across CIMMYT regional stations (namely between Mexico and sub-Saharan Africa) would be informative in optimizing collaborative breeding activities across those stations vs station-specific activities focused on local/subregional adaptation. Namely, GP (and estimates of heritability) across mega-environments would further inform the extent to which grain carotenoid traits could be treated as largely genetic parameters or whether they would need to be treated more akin to agronomic traits (for which breeding for local/subregional adaptation has been critical within provitamin A biofortification efforts; Manjeru et al. 2019). The 2 locations examined in the present study had similar Köppen–Geiger classifications (Supplementary Fig. 2), which may impact the ability to extrapolate beyond these environmental classes.

### Between-environment prediction with fewer markers

Predictive abilities were maintained between vs within environments for the 2- and 13-gene approaches, indicating that markers proximal to these genes were able to predict consistently despite environmental effects. Owens et al. (2014) tested the use of random sets of 8 genes (selected from the whole genome or from a set of 58 a priori genes for maize grain carotenoids) in prediction and found that a nonrandom set of 8 major-effect a priori genes (which were included in the 13 genes tested herein) exhibited improved performance compared to both types of random sets. That finding suggests that the non-negligible predictive abilities of the 2- and 13-gene approaches observed herein (particularly within environments, and for provitamin A traits between environments) were not solely due to gene (and corresponding marker) subsets of those sizes simply managing to sufficiently capture genomic relationships—rather, that gene identity (and namely relevance to the target traits) is important. Nonetheless, given that genome-wide markers exhibited high predictive abilities in this study, and given that GP/GS is deployed at CIMMYT for other traits (such that assaying of genome-wide markers could be beneficial for other traits as well) and such assays are increasingly low-cost, it seems that use of genome-wide markers in GS for grain carotenoid traits would be robust (with moderate to high predictive abilities observed in this study both within and between environments; with the exception of lutein between locations, which showed poor predictive abilities for all marker sets) and still resource-efficient.

### Considerations for lutein quantification and prediction in carotenoid biofortification programs

In this study, trait values for lutein in TL10 and TL11 showed slight negative correlations with β-carotene and provitamin A and slight to negligible correlations with β-cryptoxanthin and zeaxanthin in all environments (Fig. 1). While trait values for lutein in AF12 and 13 had slight to negligible correlations with β-carotene, they exhibited a slight positive correlation with provitamin A and substantially higher positive correlations (than those observed for lutein in the TL environments) with β-cryptoxanthin (median 0.45, SD 0.06) and zeaxanthin (median 0.81, SD 0.08) in all environments. These relationships would be important to monitor if seeking to increase lutein (produced in the ɑ-branch of the carotenoid pathway) alongside other carotenoid traits (namely those produced in the β-branch, including β-carotene, β-cryptoxanthin, and zeaxanthin)—particularly if selecting upon *lcyE*, which encodes the enzymatic step at the pathway branchpoint.

Interestingly, these trait relationships were consistent across years within locations. One potential explanation for the differential relationships observed between locations could be that some environmental (or repeatable GxE) factor in TL vs AF made for weaker vs stronger relationships of lutein with other traits (namely β-cryptoxanthin and zeaxanthin) in the 2 locations. While we do not exclude this possibility, it was also noted in Suwarno et al. (2015) that the HPLC method used for carotenoid quantification in TL10 and 11 allowed for better separation of lutein and zeaxanthin on the resulting chromatograms than the UPLC method used for AF12 (and the same UPLC protocol was used for AF13). Notably, the predictive abilities of GP models for lutein were poor when predicting between TL and AF environments in either direction. That finding could be due to lack of concordance between lutein as quantified via HPLC vs UPLC, as instrument platform was confounded with location. The substantially higher correlations observed between lutein and zeaxanthin in AF (compared to TL) could be artificially high due to that inferior separation, as a larger peak in the relevant range of retention times could have resulted in higher quantified levels of both compounds. Taken together, we would recommend prioritizing the prediction of values acquired through optimal analytical methods for all analytes and gathered using the same instrument platforms.

### Suitability of the TASSEL GS plugin for GP

The TASSEL GS plugin implementation of GBLUP, which has been available since 2015, is accessed through the TASSEL GUI; the results are generated quickly (in approximately 30 s in this study), and all traits can be run simultaneously. This plugin could be helpful for researchers needing to perform GP without HPC resources or who wish to test the model as part of their quantitative genetics analytical workflows (e.g. if genetic mapping is already being conducted in TASSEL) without building up dedicated custom script bases, which can represent a barrier to entry. This plugin is a user-friendly resource hosted within TASSEL, which is well documented and maintained, and could facilitate the implementation of GP/GS in breeding programs for many crops. We would also refer readers to rTASSEL (Monier et al. 2022), which offers many of the same utilities of TASSEL (including GP) in an R environment. We anticipate that both the TASSEL GUI and rTASSEL implementations could have utility for partially overlapping user bases, with the overall outcome of increased accessibility to GP methods and increased continuity with other analytical workflows routinely carried out by plant breeders/geneticists. Finally, the kin.blup() function has superceded kinship.blup() in the rrBLUP package (Endelman 2011); while both are valid from a linear algebra perspective (and are wrappers to the mixed.solve() function in the same package), kin.blup() offers the advantage of the user not needing to specify design matrices.

### Considerations for implementing GS in biofortification programs

Ten lines in this study were biofortified for β-carotene and had among the highest grain concentrations of β-carotene in the panel (Supplementary Fig. 6; Suwarno et al. 2015). Twenty iterations were conducted per method per experiment, largely to mitigate the placement of a small number of lines within a given randomized fold assignment influencing the overall median predictive ability (as calculated across iterations). The iterations with the highest predictive ability generally had low SDs between the different cross-validation folds within that iteration. The median predictive ability in most traits and environments had either no linear trend or a negative linear trend when plotted against SD, a metric for variation or dispersion, within model iterations (Supplementary Fig. 5).

Provitamin A is a “derived” trait in the sense that it was calculated as the concentration of β-carotene plus half of the concentration of β-cryptoxanthin. Given that β-cryptoxanthin is immediately downstream of β-carotene within the biosynthetic pathway, conducting GP for provitamin A itself—as a trait that is of direct interest for human nutrition—is important to biofortification efforts, and both accounts for and masks the biological complexity underlying provitamin A concentrations. *crtRB1* encodes an enzyme that catalyzes 2 consecutive steps in the carotenoid biosynthetic pathway: β-carotene to β-cryptoxanthin, and β-cryptoxanthin to zeaxanthin. This gene was found to exhibit negative pleiotropy between β-carotene and each of β-cryptoxanthin and zeaxanthin in the US maize NAM panel (Diepenbrock et al. 2021). The 10 lines with a favorable allele of *crtRB1* that were included in this study had among the highest levels of β-carotene, but their levels of β-cryptoxanthin were distributed throughout those of the rest of the population (Supplementary Fig. 6). Use of GP, whether using genome-wide markers or markers proximal to a larger set of genes, could help in optimization of provitamin A concentrations as the sum of the 2 compound concentrations without solely operating upon their direct substrate-to-product relationship, which is itself upstream of another health-beneficial carotenoid: zeaxanthin.

### Future directions and recommendations

Next steps for this work include the testing of grain carotenoid concentrations (and GP models predicting them) across mega-environments. Notably, we would recommend ensuring that the same instrument platform was used for carotenoid quantification when conducting single-trait predictions across environments, particularly for lutein. If UPLC methods that were specifically optimized for provitamin A carotenoids are being used, multi-trait GP models (based on levels of provitamin A compounds) may have higher predictive abilities for lutein (and perhaps secondarily, zeaxanthin) than single-trait models that train and predict breeding values for those non-provitamin A compounds on data from different analytical platforms. However, such approaches would be limited by the extent of the genetic correlations between levels of provitamin A and non-provitamin A compounds (which could also change as 1 or both trait sets are being selected upon) and would need to be tested empirically.

Another key next step for this work is the use of GP methods in selection. Given that the CAM panel is composed of key inbreds for the breeding program, the recurrent selection scheme proposed by Windhausen et al. (2012) for application of GP in closed populations could be pertinent. Namely, recurrent selection (repeated cycles of selection with intermating of selected individuals, in distinct rather than overlapping rounds; Labroo and Rutkoski 2022) could be used to conduct population improvement for priority traits and select candidate pre-commercial lines out of that population. Key inbreds from outside of the CAM panel could also be incorporated to help maintain genetic variation and integrate complementary favorable genomic regions (de Paula et al. 2020). We would recommend using GP methods that penalize or monitor for loss of genetic diversity; Ozimati et al. (2019) did not observe such a loss when carrying out GS for 4 traits including dry matter content in cassava, though that program prioritized crosses across (rather than within) population clusters based on genomic relationships. The TASSEL GS plugin can be readily tested by interested parties on the TASSEL tutorial data, which are included in the TASSEL installation directory, and/or on the input datasets used in the present study. In conclusion, the high predictive abilities of GP methods for maize grain carotenoid traits herein, even via less computationally intensive methods, and the availability of the TASSEL GS plugin represent the predictive ability of and an opportunity for the use of GP/GS in biofortification alongside other crop improvement efforts.

## Supplementary Material

jkae044_Supplementary_Data

## Data Availability

Genotypic and phenotypic data are available through the CIMMYT Dataverse repository using the standard data terms, with a permanent link: https://hdl.handle.net/11529/10549016 (LaPorte et al. 2024). Scripts are publicly available as Supplementary material on GitHub via https://zenodo.org/doi/10.5281/zenodo.10655189. Supplemental material available at G3 online.

## Disclaimer

This report was prepared as an account of work sponsored by an agency of the United States Government. Neither the United States Government nor any agency thereof, nor any of their employees, makes any warranty, express or implied, or assumes any legal liability or responsibility for the accuracy, completeness, or usefulness of any information, apparatus, product, or process disclosed, or represents that its use would not infringe privately owned rights. Reference herein to any specific commercial product, process, or service by trade name, trademark, manufacturer, or otherwise does not necessarily constitute or imply its endorsement, recommendation, or favoring by the United States Government or any agency thereof. The views and opinions of authors expressed herein do not necessarily state or reflect those of the United States Government or any agency thereof.
