# Supplementary material for: Investigating genomic prediction strategies for grain carotenoid traits in a tropical/subtropical maize panel
Source: G3 (Bethesda). 2024 Mar 1;14(5):jkae044. doi: 10.1093/g3journal/jkae044 (PMC11075567; doi:10.1093/g3journal/jkae044)
Supplement: jkae044_Supplementary_Data [file jkae044_supplementary_data.docx]

### Supplementary Figures and Table:


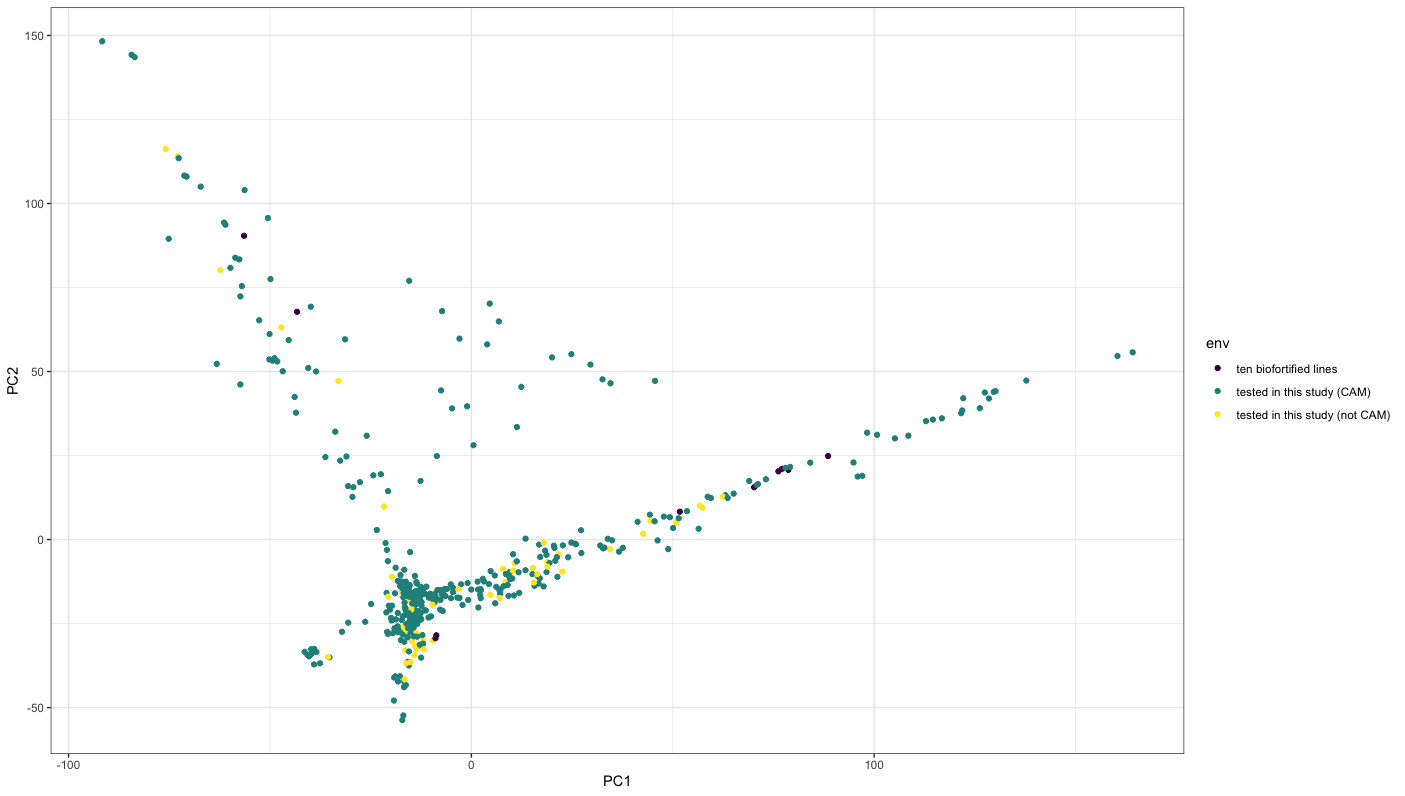


**Figure S1** PCA biplot of the genomic information from each line included in this study. The ten biofortified lines listed are part of the CAM panel. The lines included in this graph that are not part of the CAM panel (as it was described in Suwarno et al. 2015) were grown in at least one of the experimental sites, and were phenotyped with the CAM panel.


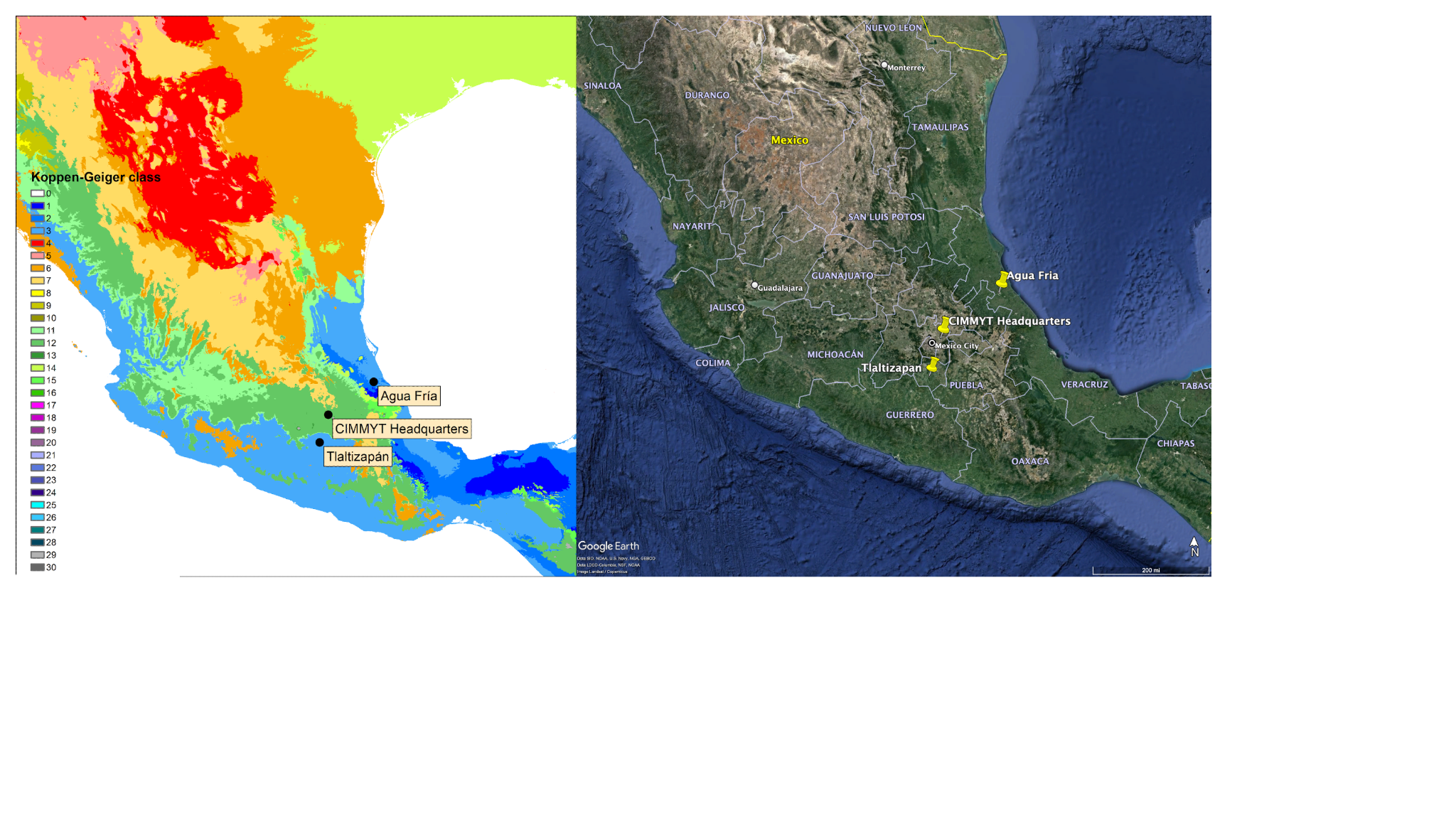


**Figure S2** The Köppen-Geiger classification of the geographic region of the field locations: Tlatizapán (TL) and Agua Fría (AF), in relation to the CIMMYT Headquarters.


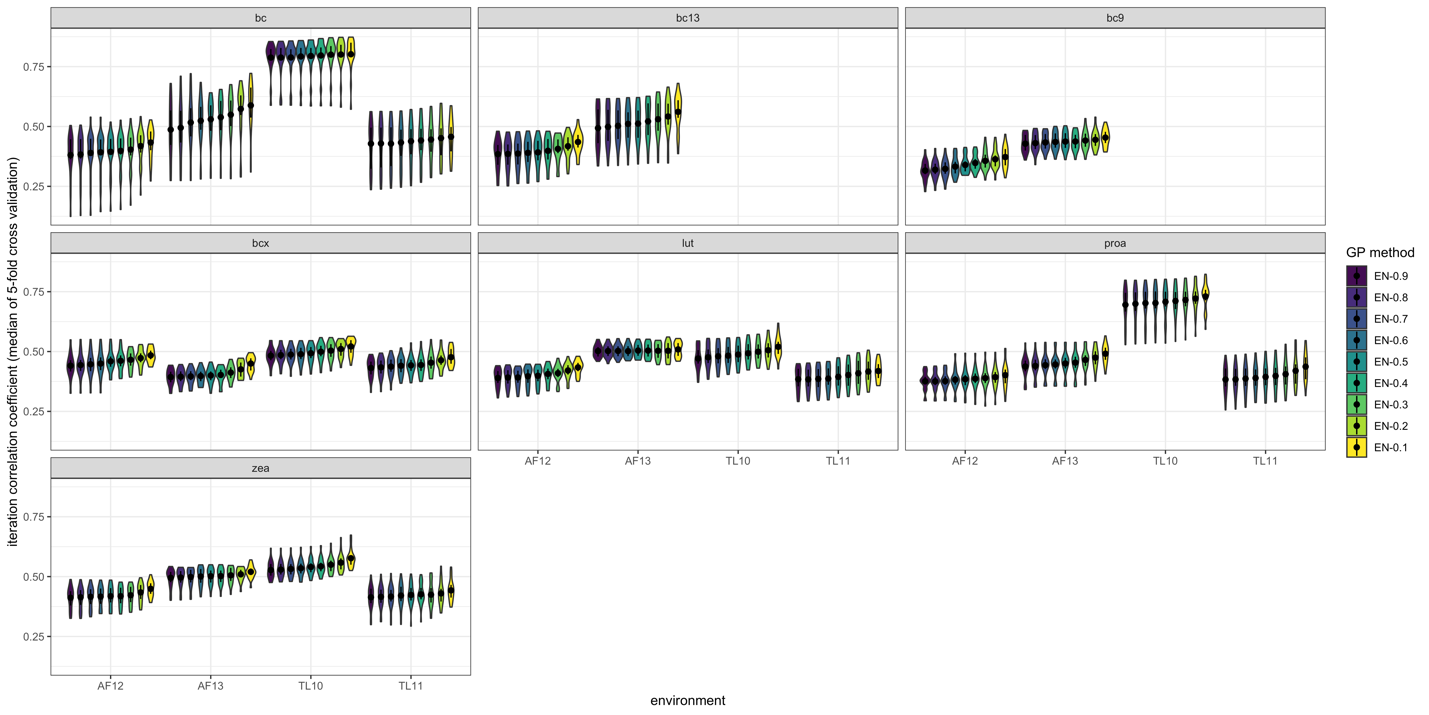


**Figure S3** Comparison of Elastic Net (EN) alpha values. The numeric values 0.1-0.9 refer to the alpha value used in each EN model.


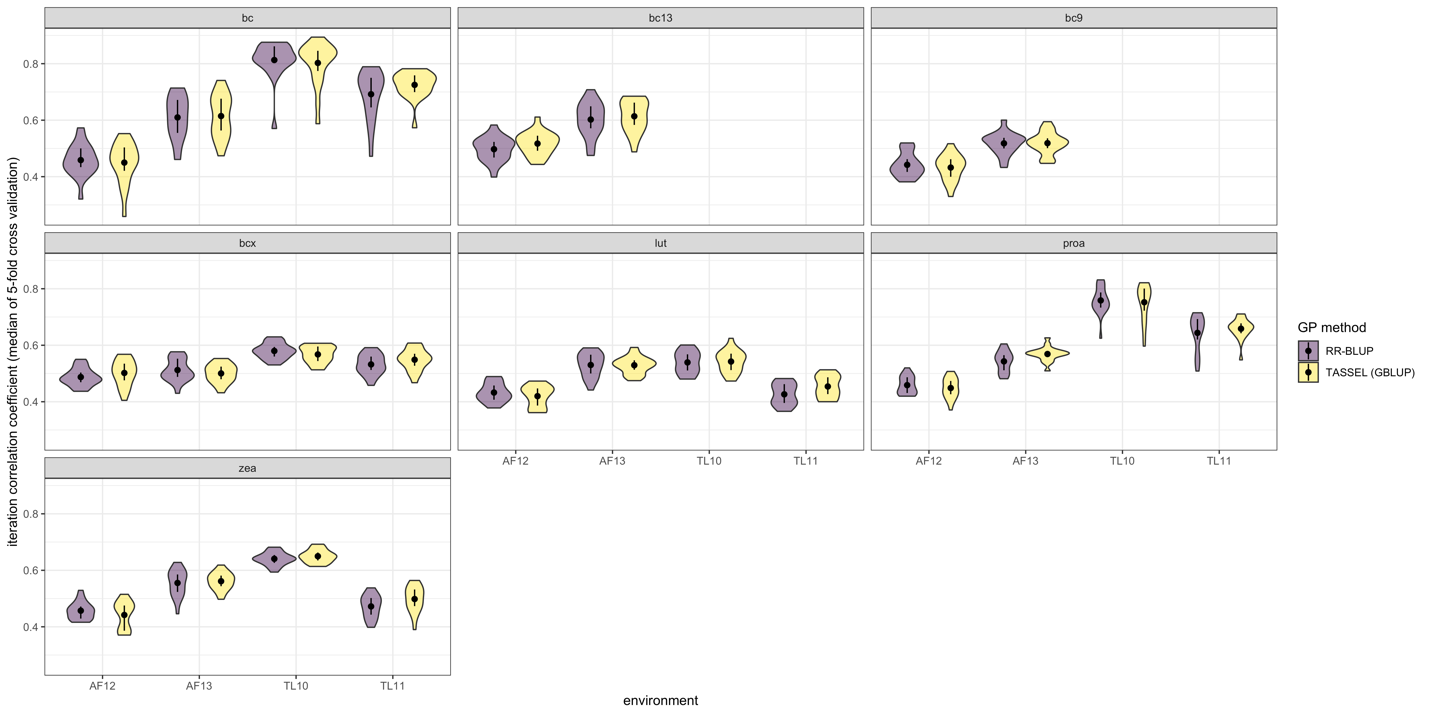


**Figure S4** Comparison of RR-BLUP and GBLUP conducted through the TASSEL Graphical User Interface Genomic Selection Plugin.


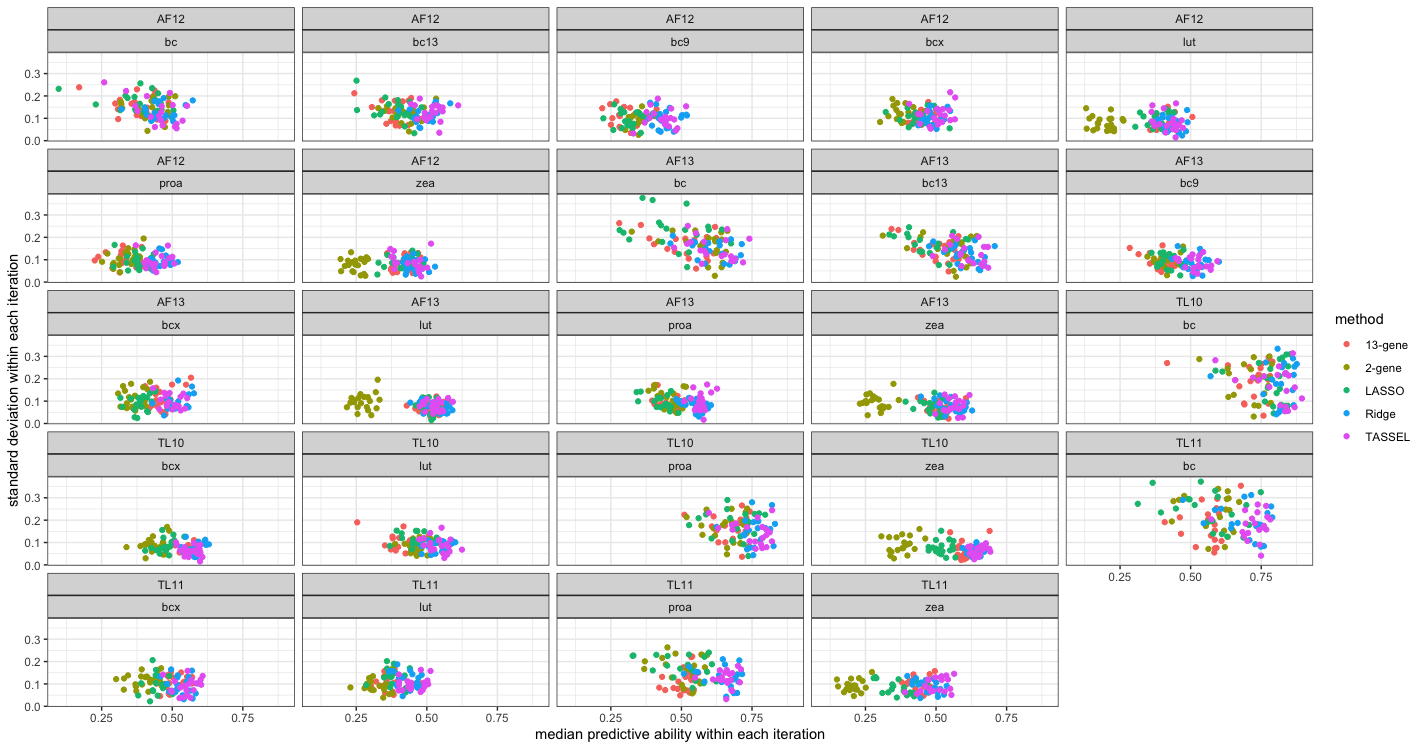


**Figure S5** Median predictive ability of each iteration vs. the standard deviation of predictive ability across folds within that iteration, for RR-BLUP in each trait and environment in this study. Environments are labeled Agua Fría 2012, Agua Fría 2013, Tlaltizapan 2010, and Tlaltizapan 2011 (AF12, AF13, TL10, and TL11, respectively). Traits are labeled as bc, β-carotene; bc9, 9-*cis*-β-carotene, bc13, 13-*cis*-β-carotene; bcx, β-cryptoxanthin; lut, lutein; proa, provitamin A; zea, zeaxanthin.

**Table S1** List of genes included in the 13-gene subset that was used in prediction. Columns are the RefGen_v4 gene ID, RefGen_v4 annotation and abbreviation, RefGen_v2 gene ID, chromosome number (Chr.; consistent across RefGen_v2 and RefGen_v4), and start bp and end bp in RefGen_v2 coordinates.

| RefGen_v4 ID | Annotated Gene Function | RefGen_v2 ID | Chr. | Start | End |
| --- | --- | --- | --- | --- | --- |
| Zm00001d027936 | phytoene desaturase 1 (*vp5*) | GRMZM2G457127 | 1 | 17657679 | 17658741 |
|  |  | GRMZM2G548584 | 1 | 17660282 | 17660393 |
|  |  | GRMZM2G410515 | 1 | 17660941 | 17667054 |
|  |  | GRMZM5G815358 | 1 | 17668483 | 17670551 |
| Zm00001d029822 | epsilon ring hydroxylase (*lut1*) | GRMZM2G143202 | 1 | 86838334 | 86848726 |
| Zm00001d001909 | plastid alternative oxidase (*im1*) | AC190835.3_FG001 | 2 | 2782695 | 2783462 |
|  |  | GRMZM2G102349 | 2 | 2782780 | 2786289 |
| Zm00001d003512/ Zm00001d003513 | zeaxanthin epoxidase 1 (*zep1*) | GRMZM2G127139 | 2 | 44440299 | 44449237 |
|  |  | GRMZM2G127139 | 2 | 44440299 | 44449237 |
| Zm00001d036345 | phytoene synthase 1 (*psy1*) | GRMZM2G300348 | 6 | 82017148 | 82021007 |
|  |  | GRMZM2G000376 | 6 | 82021510 | 82031450 |
| Zm00001d019060 | deoxy xylulose synthase 2 (*dxs2*) | GRMZM2G493395 | 7 | 14077852 | 14081075 |
|  |  | GRMZM5G836845 | 7 | 14078450 | 14079504 |
| Zm00001d011210 | lycopene epsilon cyclase (*lcyE*) | GRMZM2G012966 | 8 | 138882594 | 138889812 |
|  |  | GRMZM2G318121 | 8 | 138887488 | 138889594 |
| Zm00001d045383 | deoxy xylulose synthase 3 (*dxs3*) | GRMZM2G173678 | 9 | 20462041 | 20464497 |
|  |  | GRMZM2G173641 | 9 | 20462059 | 20467072 |
| Zm00001d048373 | carotenoid cleavage dioxygenase 1 (*ccd1*) | GRMZM2G057243 | 9 | 152086899 | 152092882 |
| Zm00001d048469 | beta carotene hydroxylase 5 (*hyd5*) | GRMZM2G382534 | 9 | 153692212 | 153694576 |
| Zm00001d026056 | beta carotene hydroxylase 1 (*crtrb1*) | GRMZM2G152135 | 10 | 136057100 | 136060219 |
| Zm00001d051458 | methyl erythritol cyclodiphosphate synthase 1 (*mecs1*) | GRMZM5G835542 | 4 | 155830779 | 155832786 |
|  |  | GRMZM2G118693 | 4 | 155833063 | 155837916 |


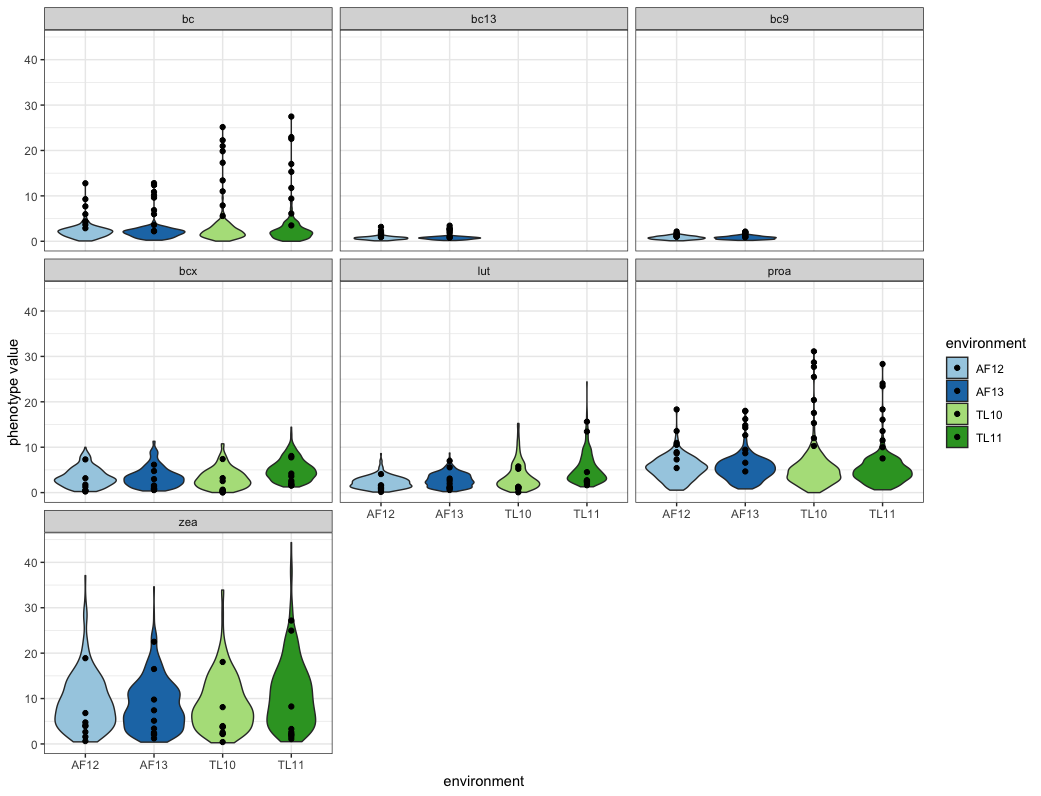


**Figure S6** The distribution of phenotypes for each trait and environment in this study, with the values of the 10 biofortified lines represented with a black dot. Environments are labeled as: Agua Fría 2012, Agua Fría 2013, Tlaltizapan 2010, Tlaltizapan 2011 (AF12, AF13, TL10, TL11, respectively), and traits are labeled as: bc, β-carotene; bc9, 9-*cis*-β-carotene; bc13, 13-*cis*-β-carotene; bcx, β-cryptoxanthin; lut, lutein; proa, provitamin A; zea, zeaxanthin.


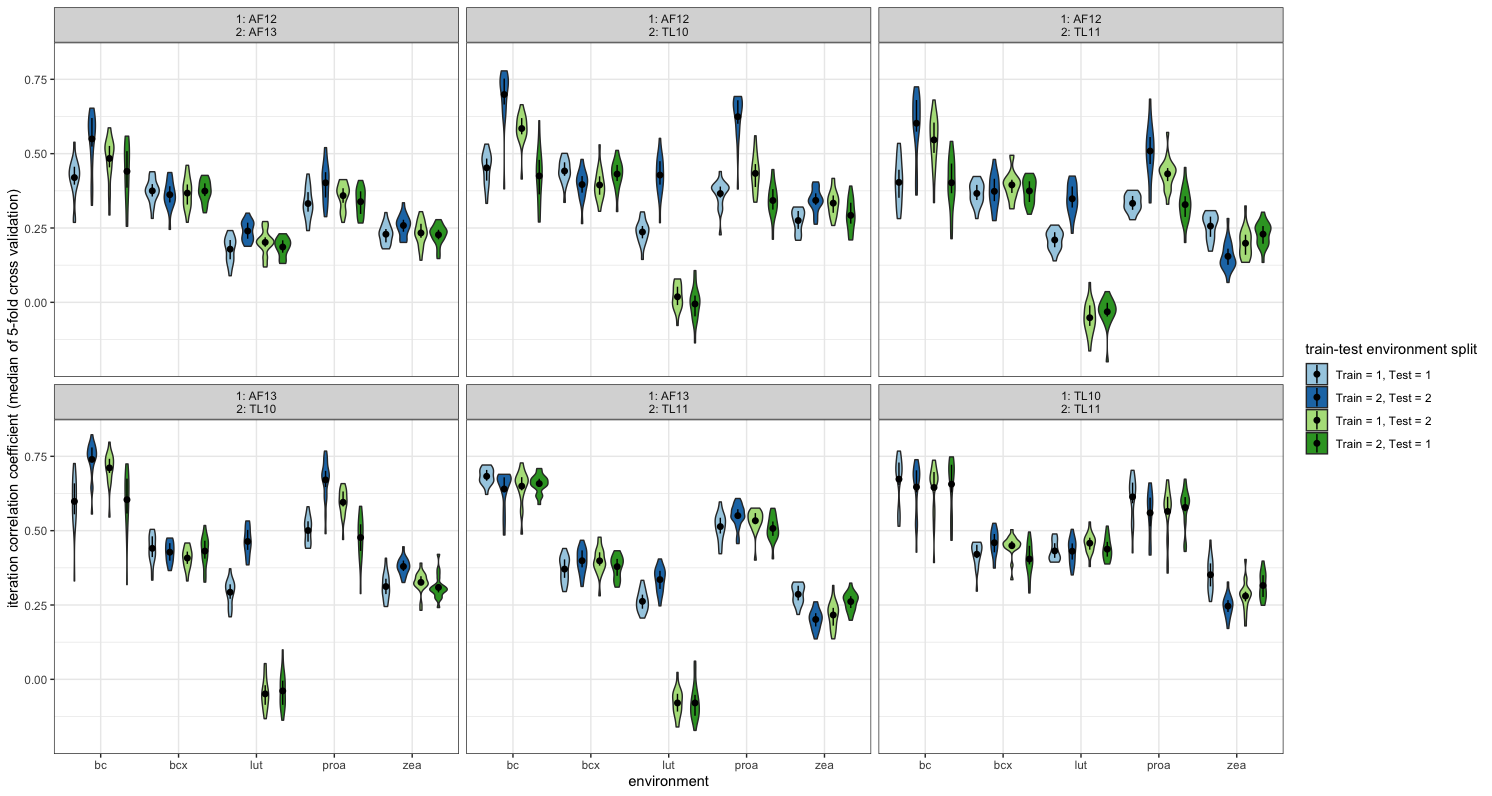


**Figure S7** Between-environment predictive ability using the two-gene RR-BLUP approach for all traits, within every pairwise combination of environments. Trait abbreviations: bc, β-carotene; bc9, 9-cis-β-carotene, bc13, 13-cis-β-carotene; bcx, β-cryptoxanthin; lut, lutein; proa, provitamin A; zea, zeaxanthin.


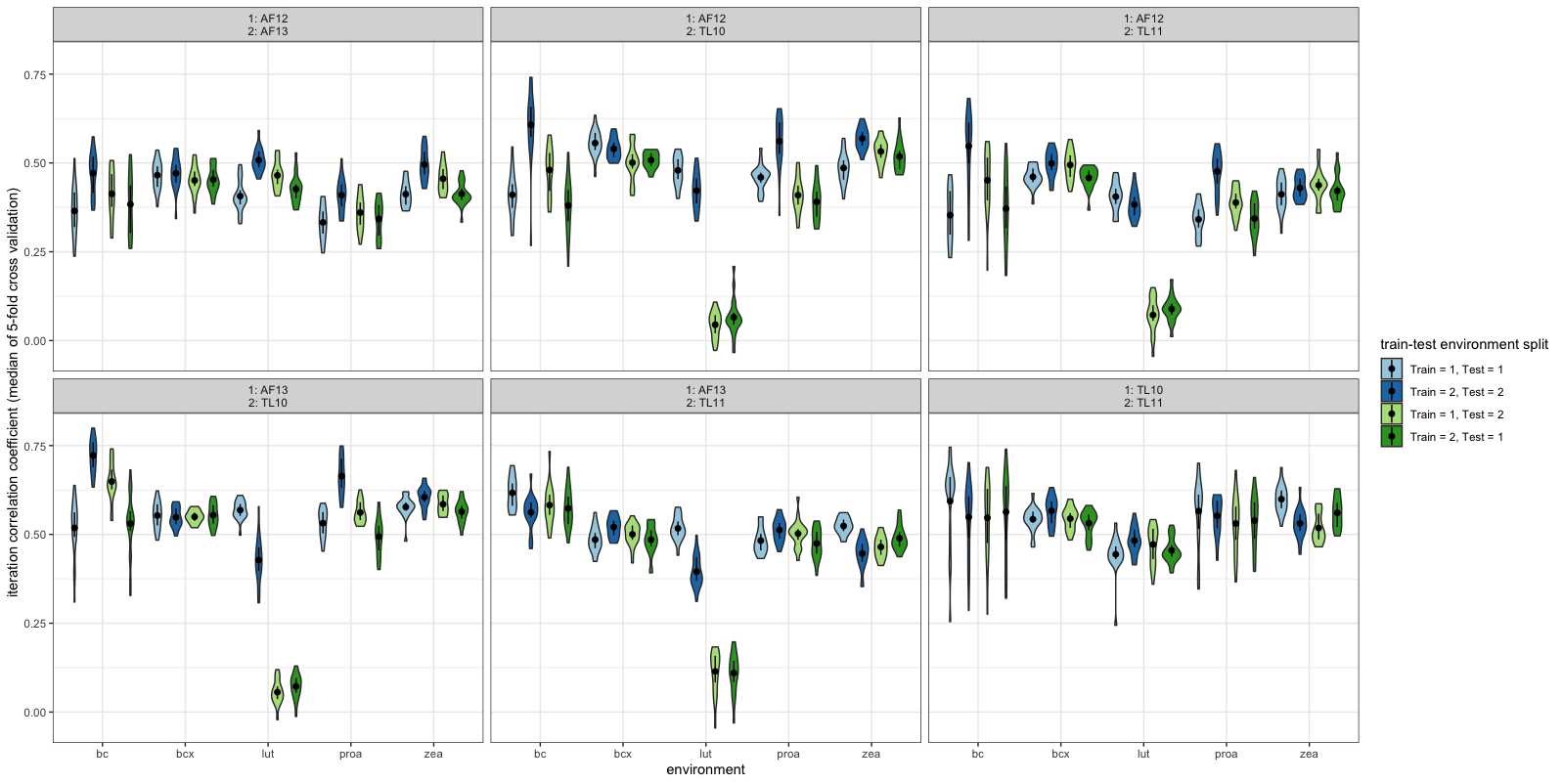


**Figure S8** Between-environment predictive ability using the 13-gene RR-BLUP approach for all traits, within every pairwise combination of environments. Trait abbreviations: bc, β-carotene; bc9, 9-cis-β-carotene, bc13, 13-cis-β-carotene; bcx, β-cryptoxanthin; lut, lutein; proa, provitamin A; zea, zeaxanthin.
